# Microscopic surgery for the repair of painful varicocele- efficacy and predictors of successful outcomes

**DOI:** 10.1186/s12610-025-00277-y

**Published:** 2025-08-15

**Authors:** Shayel Bercovich, Hen Hendel, Yossi Ventura, Shay Golan, Shachar Aharony, Ohad Shoshany

**Affiliations:** 1 Urology Department, Rabin Medical Center, Beilinson Hospital, Petach Tikva, Israel; 2 Tel Aviv Faculty of Medicine, Tel Aviv University, Tel Aviv, Israel

**Keywords:** Varicocele, Microsurgery, Scrotal pain, varicocèle, microchirurgie, douleur scrotale

## Abstract

**Background:**

Surgical repair can be offered to patients with scrotal pain of suspected varicocele origin. The estimated success rate of pain resolution is approximately 80%, although recent publications have been inconsistent. Predictive variables for successful outcomes remain contested. The current study aimed to evaluate the efficacy of microscopic repair in resolving varicocele pain and to identify variables that predict successful outcomes.

**Results:**

During the study period, microscopic subinguinal varicocelectomy was performed in 59 patients with varicocele-related pain. Grade III left varicocele was present in 36 (61%) patients. The median width of the left varicocele, as measured by ultrasound, was 4.2 mm (IQR 4–5), with reflux identified in 39 (66%) patients. The most common type of pain—dull pain—was present in 39 (66%) patients. Exertional pain and sharp pain were reported by 11 (19%) and 9 (15%) patients, respectively. The median follow-up time was 24 months (IQR 13–35), while 46 (78%) patients were contacted by telephone more than 12 months after surgery. Fifty (85%) patients reported complete pain resolution, while partial resolution and persistent pain were reported by 4 (6%) and 5 (9%) patients, respectively. Two variables that increased the risk of pain persistence were repeat varicocele repair surgery and pain as a secondary or additional indication for surgery. Follow-up time of more than 12 months after surgery reduces the risk of pain persistence.

**Conclusions:**

A varicocelectomy is a good option for resolving painful varicocele in most patients, especially those whose only indication for surgery is pain. Repeat varicocele surgery appears to increase the risk of persistent pain. A longer follow-up period (more than 12 months after surgery) increases the chances of pain resolution.

## Résumé

**Contexte:**

La réparation chirurgicale peut être proposée aux patients qui présentent des douleurs scrotales suspectées d’être dues à une varicocèle. Le taux de réussite estimé de résolution de la douleur est d’environ 80 %, bien que des publications récentes aient été contradictoires. Les variables prédictives de résultats positifs restent contestées. L’étude actuelle visait à évaluer l’efficacité de la réparation microscopique dans la résolution de la douleur due à une varicocèle, et à identifier les variables prédictives de résultats positifs.

**Résultats:**

Au cours de la période d’étude, une varicocélectomie microscopique sub-inguinale a été réalisée chez 59 patients qui souffraient de douleurs liées à la varicocèle. Une varicocèle gauche de grade III était présente chez 36 patients (61 %). La largeur médiane de la varicocèle gauche, mesurée par échographie, était de 4,2 mm (IQR 4-5), avec un reflux identifié chez 39 patients (66 %). Le type de douleur le plus courant - une douleur sourde - était présent chez 39 patients (66 %). La présence de douleurs à l’effort et de douleurs aiguës ont été signalées respectivement par 11 (19 %) et 9 (15 %) patients. La durée médiane du suivi fut de 24 mois (IQR 13-35), alors que 46 patients (78 %) ont été contactés par téléphone plus de 12 mois après l’opération. Cinquante patients (85 %) ont signalé une résolution complète de la douleur, mais une résolution partielle et une douleur persistante ont été signalées respectivement par 4 (6 %) et 5 (9 %) patients. Deux variables augmentaient le risque de persistance de la douleur : la chirurgie réparatrice répétée de la varicocèle, et la douleur comme indication secondaire ou supplémentaire de la chirurgie. Un temps de suivi de plus de 12 mois après la chirurgie réduit le risque de persistance de la douleur.

**Conclusions:**

Une varicocélectomie est une bonne option pour résoudre la varicocèle douloureuse chez la plupart des patients, en particulier ceux dont la seule indication pour la chirurgie est la douleur. La chirurgie répétée d’une varicocèle semble augmenter le risque de douleur persistante. Une période de suivi plus longue (plus de 12 mois après la chirurgie) augmente les chances de résolution de la douleur.

## Introduction

Varicocele is a common anatomical anomaly characterized by the dilation and tortuosity of the veins in the pampiniform plexus. The prevalence of varicocele in the general population ranges from approximately 5% to 15% but increases to 25% to 40% in men who have primary infertility [1, 2]. Research on varicocele has mainly focused on its association with male infertility. However, varicocele is also associated with chronic pain in a considerable number of men. The prevalence of painful varicocele ranges from 2 to 10% among men experiencing chronic testicular pain [3–5]. The pain associated with varicocele is often described as a dull ache or a feeling of heaviness, which may worsen after prolonged standing, sitting, or physical exertion [3]. Most patients report pain relief when lying down [6]. Although there are various techniques for conservative treatment of painful varicocele, in cases where such treatment is ineffective, varicocele ligation surgery is suggested [5, 7]. 

Several studies published in previous decades investigated the effectiveness of surgery for treating pain associated with varicocele. Early studies reported resolution of scrotal pain in approximately 50–60% of men who underwent varicocele repair, while more contemporary series report pain resolution as high as 80–90% [5, 8, 9]. However, only a small number of these studies reported the use of advanced techniques such as subinguinal ligation or microsurgery[5, 10–13], and most lacked long follow up periods [5, 11]. Successful surgical outcomes appear to be associated with multiple variables, including pain characteristics, duration of pain before surgery, the grade of the varicocele, and the selected surgical approach [5, 14, 15].

The current study aims to evaluate the success of subinguinal microscopic varicocele ligation surgery for pain treatment and to determine predictive variables associated with postoperative pain relief. The research focuses on identifying patients who are most likely to benefit from surgical intervention and aims to improve clinical outcomes.

## Patients and methods

For this retrospective study, a systematic telephone follow-up was used to collect data from patients who had undergone microsurgical varicocele repair for a primary complaint of scrotal pain between 2018 and 2023. All patients were operated on by the same surgical team using a subinguinal approach under a surgical microscope. The study was approved by the medical center's ethics committee, and all participants provided informed consent (RMC-0747–22).

**Fig. 1:**
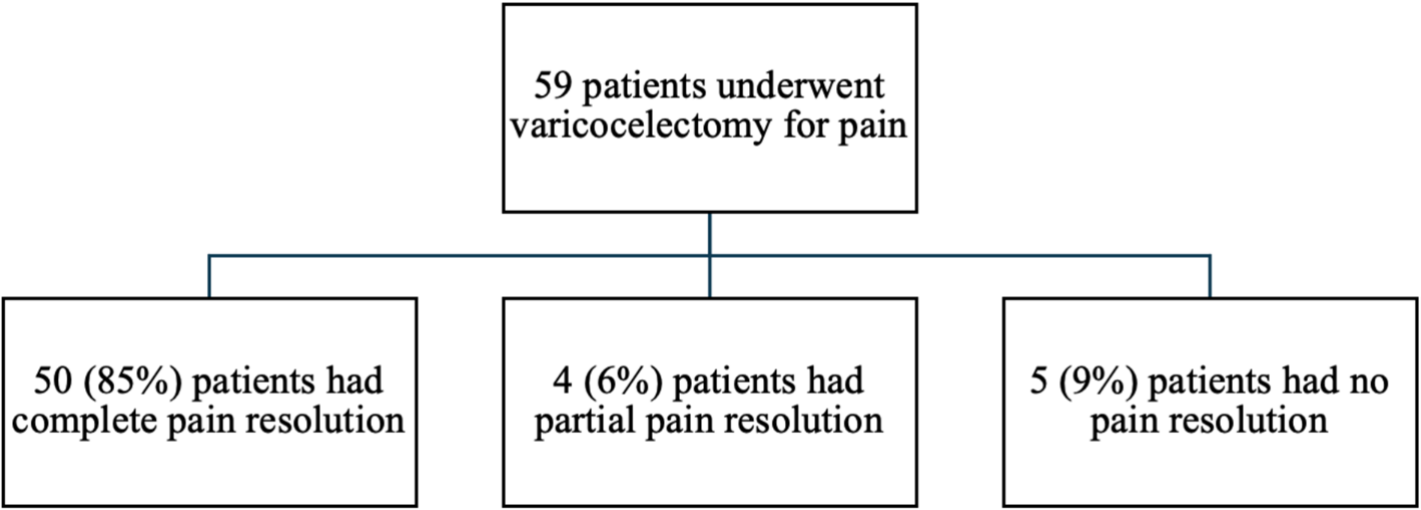
Flowchart depicting study inclusion for surgical intervention, follow-up, and outcomes of pain resolution in patients undergoing subinguinal microsurgical varicocelectomy for scrotal pain

The study included men aged 18 to 40 years who underwent varicocele repair surgery for chronic pain. Chronic pain was defined as pain lasting for at least six months, unresponsive to conservative treatment (rest, anti-inflammatory medications, tight-fitting underwear for scrotal support), and with no evidence of an alternative cause. Differential diagnoses for hernia, tendinitis, epididymitis, or trauma were ruled out based on medical history, physical examination, and imaging when necessary Fig. 1.

Clinical evaluation before surgery included a complete medical history and thorough physical examination to rule out other etiologies of chronic orchialgia. The medical history encompassed physical activity levels, work history, a detailed description of pain characteristics (location, nature, radiation, frequency, duration, and severity), and other pain complaints such as back pain or ejaculatory pain, as well as sexual, lower urinary symptoms, and bowel symptoms. The physical examination included palpation of the scrotal contents in both standing and supine positions, including assessment with the Valsalva maneuver, and grading of varicoceles according to standard classification (grades 1–3). The groin was evaluated for lymphadenopathy, hernia, and tenderness of various tendons (rectus abdominis insertion, adductor tendon, and inguinal tendon). The abdomen was palpated for superficial tenderness. Demographic data and imaging results (primarily Doppler ultrasonography) were also collected. When another etiology for the chronic orchialgia was suspected, surgery was postponed until a complete evaluation and treatment by the relevant specialists (e.g., general surgeons, orthopedists, or pain clinic specialists) had been completed.

All surgeries were performed using a subinguinal microsurgical approach as described by Goldstein et al.[16] The spermatic cord was elevated through a subinguinal incision. The testis was delivered and gubernacular and external pudendal vessels were dissected and ligated. Cremasteric And spermatic veins were identified, dissected and ligated using microsurgical instruments. A doppler probe was utilized to identify and preserve the testicular artery/arteries, and lymphatics were preserved as well.

Following surgery, a structured telephone follow-up was conducted by a medical survey who was not part of the operating team, using a standardized questionnaire to assess outcomes. The minimum follow-up period was six months. Preoperative pain characteristics and duration were confirmed, and changes in pain following surgery were comprehensively evaluated. Each patient was asked whether the pain had completely resolved, improved, or remained unchanged.

### Statistical analyses

Data were analyzed using SPSS software version 27. Categorical variables were summarized by number and percentage, and continuous variables by median and interquartile range (IQR). Multivariate tests, including logistic regression, were used to examine associations between preoperative characteristics and surgical success. Statistical significance was defined as *P* ≤ 0.05.

## Results

### Participants

The study included 59 patients who underwent microsurgical varicocele ligation for scrotal pain between 2018 and 2023. The median age of the patients was 22 years (interquartile range [IQR]: 20–28), and the median BMI was 23 kg/m^2^ (IQR: 20.5–24.9). Four patients (6%) had previously undergone varicocele surgery (Table 1).

**Table 1:** Demographics and characteristics of patients with painful varicocele

Characteristic	No
Total No. of patients (%)	59 (100)
Age (years), median (IQR)	22 (20–28)
BMI, median (IQR)	23 (20.5–24.9)
2nd varicocele operation (%)	4 (6)
Indication for surgery	
Pain only (%)	15 (26)
Pain & Infertility (%)	44 (74)
Varicocele grade	
Grade 1 (%)	2 (3)
Grade 2 (%)	21 (36)
Grade 3 (%)	36 (61)
Bilateral varicocele (%)	6 (10)
Varicocele size by US (mm), median (IQR)	4.2 (4–5)
Varicocele with reverse flow (%)	39 (66)
Pain type	
Dull (%)	39 (66)
Exercise related (%)	11 (19)
Sharp (%)	9 (15)
Pain duration (months), median (IQR)	10 (7–13)
Lower testicular volume (%)	24 (41)
Abnormal sperm analysis (%)	39 (66)
Follow up (months), median (IQR)	24 (13–35)
Follow up duration	
< 12 months (%)	13 (22)
> 12 months (%)	46 (78)
Pain resolution	
Complete (%)	50 (85)
Partial (%)	4 (6)
Not resolved (%)	5 (9)
Complications post-OP	
Hydrocele (%)	1 (1.5)
Edema (%)	1 (1.5)
Testicular atrophy (%)	0 (0)

This table summarizes the baseline demographic, clinical, and ultrasound characteristics of the 59 patients who underwent microsurgical varicocelectomy for painful varicocele. Values are presented as number (%) or median (interquartile range, IQR). *BMI* = Body Mass Index, *IQR* = Interquartile Range, *US* = Ultrasound, post-*OP* = Postoperative

### Preoperative varicocele and pain characteristics

In 15 patients (26%), surgery was performed for pain alone, whereas in 44 patients (74%), the pain was part of a combined indication (e.g., pain in addition to infertility or testicular atrophy). Most patients (61%) were diagnosed with grade 3 varicocele, 36% with grade 2, and only 3% with grade 1. Bilateral varicocele was present in 10% of cases. The median vein diameter on ultrasonography was 4.2 mm (IQR: 4–5). Reverse flow was demonstrated in 66% of cases. The types of pain reported included dull pain (66%), exertion-related pain (19%), and sharp pain (15%). The median pain duration before surgical intervention was 10 months (IQR: 7–13). Testicular volume reduction was observed in 41% of cases, and 66% showed pathological findings in semen analysis (Table 1).

### Postoperative follow-up

The median follow-up duration was 24 months (IQR: 13–35), with 22% of patients followed for less than 12 months and 78% followed for longer. Surgical success was measured by pain resolution: 85% of patients reported complete resolution of pain, 6% reported partial improvement, and 9% experienced no improvement. The postoperative complication rate was low, with one case of hydrocele (1.5%) and one case of edema (1.5%) (Table 1).

Analysis of variables associated with unsuccessful surgical pain relief identified a second (repeat) surgery as a significant risk factor, with an odds ratio (OR) of 4.19 (95% CI: 1.08–20.29, *P* = 0.05). In contrast, having pain as the sole indication for surgery was identified as a positive predictor of a successful outcome (OR: 0.19, CI: 0.03–0.94, *P* = 0.03). A follow-up period longer than six months was also associated with a reduced risk of unsuccessful surgical outcomes (OR: 0.23, CI: 0.09–0.91, *P* = 0.02). Surgical outcomes were not associated with BMI, pain duration, pain type, varicocele grade, vein diameter on ultrasonography, or the presence of reverse flow (Table 2).

**Table 2:** Variables associated with unresolved pain after varicocelectomy

	**Odds ratio**	**95% CI**	*P*-value
﻿Age (years)	0.69	0.41–1.16	0.17
BMI	0.8	0.55–1.18	0.26
2nd operation	**4.19**	**1.08–20.29**	**0.05**
Pain only indication	**0.19**	**0.03–0.94**	**0.03**
Dull pain	0.87	0.43–1.76	0.37
Varicocele Grade 3	0.7	0.59–1.23	0.31
Varicocele size by US > 5 mm	3.18	0.37–27.07	0.28
Varicocele with reverse flow	1.6	0.45–12.3	0.66
Follow up duration > 6 months	**0.23**	**0.09–0.91**	**0.02**

This table shows the odds ratios and 95% confidence intervals for variables evaluated as predictors of persistent pain following surgery. Multivariate logistic regression analysis was performed. Statistically significant results are marked in bold (*P* < 0.05). *BMI* = Body Mass Index, *CI* = Confidence Interval, *US* = Ultrasound

## Discussion

Our study found a high success rate for microsurgical varicocelectomy in relieving scrotal pain associated with varicocele, with complete pain resolution reported in 85% of patients. This rate is consistent with reports in the literature, which cite a success range of 80%–90% for microsurgical techniques [5, 12, 17]. The subinguinal microsurgical approach used in this study has been identified in several previous studies as an effective and safe technique, associated with low rates of complications and recurrence and optimal clinical outcomes [11, 17]. In the present study, one patient developed a hydrocele, and there were no cases of testicular atrophy. These findings support the prevailing view that microsurgery, particularly the subinguinal approach, is highly effective for the treatment of painful varicocele.

One of the key findings of this study is the high success rate observed among patients who underwent surgery for pain alone, without additional indications such as infertility. This finding is also supported by the work of Punjani et al., who showed that pain as an independent variable does not predict a poor outcome and may even be a positive prognostic indicator [18]. We did not find pain quality to be a predictor of successful outcome, unlike a meta-analysis by Han et al. which found that pain quality, especially dull pain, is a significant predictor of surgical success [5].

The study also found that repeat surgery is a significant risk factor for persistent postoperative pain following varicocelectomy. Among the four patients who underwent a second operation, the initial surgery was not always performed microscopically. In two cases, based on surgical reports and patient history, the first procedure involved a non-microsurgical technique, likely associated with limited visualization. During the subsequent microsurgical subinguinal operations performed by our team, we frequently identified small or deep collateral veins, particularly cremasteric or external spermatic veins, that were likely missed during the primary procedure. This finding aligns with prior literature indicating that the success rates of secondary varicocele repairs are generally lower than those of primary procedures. One primary explanation for this phenomenon is the presence of postoperative scar tissue, which can obscure anatomical landmarks and complicate surgical dissection, thereby increasing the likelihood of incomplete vein ligation or iatrogenic injury [17]. Additionally, recurrent varicoceles often involve collateral or aberrant venous pathways that were not ligated during the initial surgery. This makes subsequent repairs technically more challenging and prone to failure [19]. Marmar et al. also note that recurrence after primary surgery may stem from deep pelvic veins or collateral channels, which are difficult to identify without meticulous dissection or specialized techniques [19]. These observations underscore the importance of employing precise techniques during the initial surgery to minimize the need for revision procedures and optimize long-term outcomes.

Another noteworthy finding is the positive association between longer follow-up duration (more than 6 months) and pain relief. This finding was not reported by previous studies, most of which had a shorter follow-up period of 3–6 months, which may have missed this long-term effect. Our findings suggest that the benefits of surgery are sustained and may continue to improve over time. This outcome underscores the importance of extended follow-up in evaluating the full impact of the intervention, as also noted by Owen et al.^12^. The notion that chronic pain may require a longer period of time to improve after surgery is also supported by literature on microsurgical testicular denervation for chronic orchialgia. While most studies reported only median follow up periods to pain resolution, some suggest that pain resolution may increase with longer follow up time – in some cases, more than 6 months. [20–22]

Consistent with the conclusions of Han et al., the current study also found no significant association between varicocele grade and surgical outcomes. This absence of correlation extended to venous diameter and retrograde flow parameters. While the cessation of reflux is a primary mechanism for symptom relief, other mechanisms may contribute as well- such as reduction in venous congestion, alleviation of local inflammatory responses, and possible interruption of nerve fibers around the spermatic cord (analogous to a partial denervation effect). These finding supports the current understanding that surgical decision-making should not be based solely on varicocele parameters but on a combination of clinical features, including pain quality, duration, and its impact on quality of life [5].

While our study supports the efficacy of subinguinal microsurgical varicocelectomy for treating painful varicocele, it is important to acknowledge that other therapeutic modalities also exist and have demonstrated comparable outcomes in selected patients. Endovascular embolization, in particular, has emerged as a minimally invasive alternative to surgical repair. Several studies report success rates for pain relief ranging from 70 to 85%, comparable to those observed with surgery, especially in centers with high procedural expertise [23, 24]. Embolization offers the advantages of shorter recovery time, local anesthesia, and no incisions, though it may be associated with higher recurrence rates in some series and depends heavily on operator skill and vascular anatomy [24].

In addition to embolization, alternative surgical techniques—including laparoscopic ligation and high retroperitoneal Palomo procedures—have also been used to treat painful varicocele, though most comparative studies suggest that microsurgical approaches offer lower complication and recurrence rates [11, 17]. Thus, while microsurgical varicocelectomy remains a widely utilized and effective strategy, particularly when performed by experienced surgeons, treatment should be individualized. The choice of modality should be based on institutional availability, operator expertise, patient preference, and specific anatomical considerations.

### Limitations of the study

Our study had several limitations. First, this was a retrospective study based on telephone follow-up data and, therefore, susceptible to recall bias. Second, objective data such as standardized pain scores or routine postoperative imaging were not consistently collected from all patients. Additionally, the sample size was limited and drawn from a single medical center. Lastly, we believe patient selection is of utmost importance for achieving a high success rate and may be underreported in studies evaluating postoperative pain resolution.

## Conclusions

Our findings support the effectiveness of microsurgical varicocelectomy as a successful treatment for scrotal pain caused by varicocele, particularly when pain is the sole indication for surgery. Repeat surgery was identified as a risk factor for persistent pain, while long-term follow-up and pain as a sole indication were associated with positive outcomes. Further large-scale prospective studies are needed to identify additional precise prognostic factors and to establish personalized treatment approaches.

## Data Availability

No datasets were generated or analysed during the current study.

## Abbreviations

IQR: Interquartile range
OR: Odds ratio
CI: Confidence interval
BMI: Body mass index
US: Ultrasound
